# New insights into glioma frequency maps: From genetic and transcriptomic correlate to survival prediction

**DOI:** 10.1002/ijc.34336

**Published:** 2022-11-24

**Authors:** Hongbo Bao, Peng Ren, Liye Yi, Zhonghua Lv, Wencai Ding, Chenlong Li, Siyang Li, Zhipeng Li, Xue Yang, Xia Liang, Peng Liang

**Affiliations:** ^1^ Department of Neurosurgery Harbin Medical University Cancer Hospital Harbin China; ^2^ Laboratory for Space Environment and Physical Science Harbin Institute of Technology Harbin China; ^3^ School of Life Science and Technology Harbin Institute of Technology Harbin China; ^4^ Department of Neurosurgery The Second Affiliated Hospital of Harbin Medical University Harbin China; ^5^ Department of Neurology The First Affiliated Hospital of Harbin Medical University Harbin China; ^6^ Department of Information Harbin Medical University Cancer Hospital Harbin China

**Keywords:** frequency map, GBM, LGG, survival prediction, synapse

## Abstract

Increasing evidence indicates that glioma topographic location is linked to the cellular origin, molecular alterations and genetic profile. This research aims to (a) reveal the underlying mechanisms of tumor location predilection in glioblastoma multiforme (GBM) and lower‐grade glioma (LGG) and (b) leverage glioma location features to predict prognosis. MRI images from 396 GBM and 190 LGG (115 astrocytoma and 75 oligodendroglioma) patients were standardized to construct frequency maps and analyzed by voxel‐based lesion‐symptom mapping. We then investigated the spatial correlation between glioma distribution with gene expression in healthy brains. We also evaluated transcriptomic differences in tumor tissue from predilection and nonpredilection sites. Furthermore, we quantitively characterized tumor anatomical localization and explored whether it was significantly related to overall survival. Finally, we employed a support vector machine to build a survival prediction model for GBM patients. GBMs exhibited a distinct location predilection from LGGs. GBMs were nearer to the subventricular zone and more likely to be localized to regions enriched with synaptic signaling, whereas astrocytoma and oligodendroglioma tended to occur in areas associated with the immune response. Synapse, neurotransmitters and calcium ion channel‐related genes were all activated in GBM tissues coming from predilection regions. Furthermore, we characterized tumor location features in terms of a series of tumor‐to‐predilection distance metrics, which were able to predict GBM 1‐year survival status with an accuracy of 0.71. These findings provide new perspectives on our understanding of tumor anatomic localization. The spatial features of glioma are of great value in individual therapy and prognosis prediction.

## Graphical abstract

What's new?

The spatial distribution of glioma is nonrandom. However, whether pathological grade influences glioma distribution across the brain remains unclear. Here, the authors built a population‐based frequency map for 396 patients with glioblastoma multiforme and 190 patients with lower‐grade glioma, revealing their distinct topographic localization patterns. Further results suggested that neuron and glioma synaptic interactions and electrochemical communications were involved in determining glioblastoma multiforme location, bringing a new perspective on glioma location predilection and gliomagenesis. Moreover, based on the identified tumor location features, the authors built a survival prediction model for patients with glioblastoma multiforme.

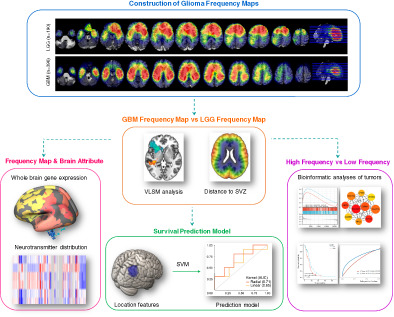

**Abbreviations**
GBM: glioblastoma multiforme
LGG: lower‐grade glioma
OS: overall survival
ROI: region of interest
SVM: support vector machine
SVZ: subventricular zone
TME: tumor microenvironment
VLSM: voxel‐based lesion‐symptom mapping
WHO: World Health Organization

## 1 INTRODUCTION

Glioma is the most aggressive primary CNS tumor in adults.^1^ Despite advances in early diagnosis and comprehensive treatments, the prognosis of glioma patients remains poor. New insights into gliomagenesis and more individualized therapeutic approaches may facilitate the development of novel therapies and benefit more patients.

Anatomic topographic location is one of the factors that affect glioma prognosis. For example, some studies found that gliomas involving the synchronous subventricular zone (SVZ) and corpus callosum presented with poor overall survival (OS).^2^, ^3^ With accumulating evidence, it has been widely believed that the spatial distribution of glioma across the brain is not random. Population‐based studies demonstrate that the right hemisphere has a higher tumor frequency than the left and gliomas are more likely to be located in the frontal and temporal lobes than the other lobes.^4^ More interestingly, recent evidence has shown that glioblastoma multiforme (GBM) with different isocitrate dehydrogenase 1 mutation (IDH1) statuses, vascular endothelial growth factor (VEGF) expression levels, molecular subtypes could also harbor a spatial localization preference,^5^, ^6^, ^7^ suggesting that certain characteristics of tumors could be reflected in their anatomical location. However, whether pathologic grade could affect glioma distribution across the whole brain remains largely unclear.

Tumor grading provides underlying prognostic and thus is of critical importance to clinical care. Clinicopathological CNS tumor grading has long been linked to overall expected clinical‐biological behaviors and the ranges of grades used for tumor types have been generally retained in the newly updated fifth edition of the World Health Organization (WHO) Classification of Tumors of the Central Nervous System (WHO CNS5).^8^ According to traditional histological grading, GBM (grade IV) has a much higher recurrence and mortality than lower‐grade gliomas (LGGs, grade II‐III).^9^, ^10^ We are curious whether GBM and LGG have different spatial distribution and what mechanisms might affect tumor location predilection.

It has been suggested that the predilection sites where brain glioma preferably occurs may be dependent on its origin and stem cells. Emerging data suggest that the SVZ and the dentate gyrus of the hippocampus are neurogenic niches in the adult human brain.^11^ Neural stem cells and progenitor cell populations residing here are regarded as the cells of origin of gliomas, while oligodendrocyte precursor cells (OPCs) seem particularly involved in the origin of LGG.^12^ This partly explains why there is a higher incidence of gliomas around the temporal horns of the lateral ventricles.^5^ Nevertheless, the localization of glioma is not limited within the SVZ areas, but rather exhibits a unique distribution across the whole brain. Additional theories are needed to explain this.

The nonrandom glioma distribution may also potentially be a result of several factors, such as the tumor microenvironment (TME) and tumor migration that foster its progression.^13^ At the cellular level, immune cells, microglia, endothelial cells, astrocytes and neurons constitute the complex brain TME.^14^, ^15^ Interaction and communication with these nontumor cells could regulate glioma development and resistance to therapy.^15^ Another impacting factor is glioma migration‐infiltration, a complex, dynamic process involving regulation of cell adhesion, cytoskeletal rearrangements and modification of the extracellular environment.^13^ Together, the final distribution of glioma is a combined result of tumorgenesis, migration and interaction with the TME. Therefore, although different glioma subtypes mainly originate from the SVZ, it is tempting to speculate that the distinct progression patterns and biological features of LGG and GBM may guide tumor cells to migrate to their respective permissive microenvironment.

**FIGURE 1:**
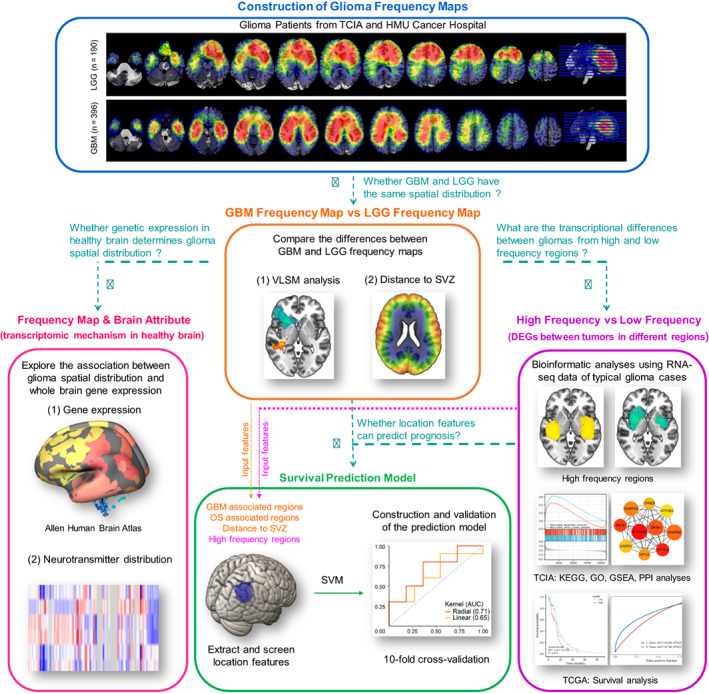
Flow diagram of study design

In our study, we aimed to investigate the spatial distribution of tumors in GBM and LGG patients, and to uncover potential underpinnings of the location preference of GBM and LGG by exploring their genetic and transcriptional correlates (Figure 1). We started by comparing the association between glioma frequency map and whole brain normal gene expression and neurotransmitter distribution, which could reflect TME and the distribution of stem cells. Next, we screened typical cases that were located in the predilection and nonpredilection regions, followed by transcriptomic analysis to reveal the interactions between tumor transcriptional features and location. Finally, we extracted quantified radiographic features from the frequency map and built a model to predict the 1‐year survival status of GBM patients.

## 2 METHODS

### 2.1 Patients and MRI image data

**FIGURE 2:**
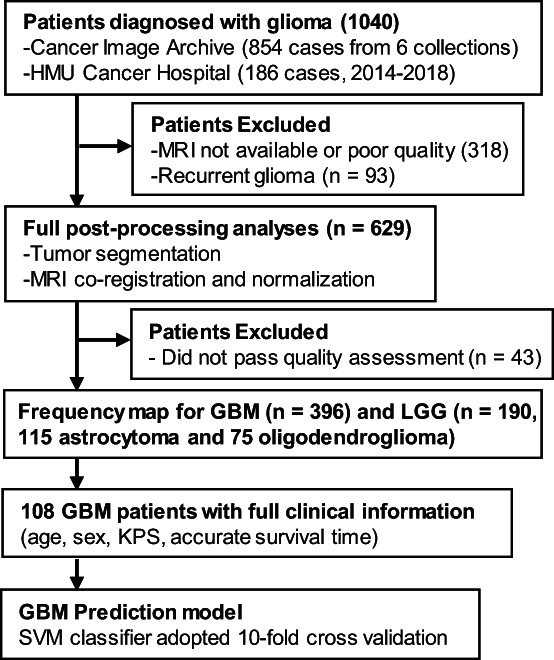
Patient selection flowchart

To construct a tumor frequency map, we collected MRI data and clinical information of glioma patients from Harbin Medical University (HMU) Cancer Hospital between 2014 and 2018 and The Cancer Imaging Archive (TCIA) (https://www.cancerimagingarchive.net).^16^ Images from TCIA were collected from different collections, including REMBRANDT (https://wiki.cancerimagingarchive.net/display/Public/REMBRANDT), TCGA‐GBM (https://wiki.cancerimagingarchive.net/pages/viewpage.action?pageId=1966258), TCGA‐LGG (https://wiki.cancerimagingarchive.net/pages/viewpage.action?pageId=5309188), Ivy GAP (https://wiki.cancerimagingarchive.net/pages/viewpage.action?pageId=22515597), QIN GBM Treatment Response (https://wiki.cancerimagingarchive.net/display/Public/QIN+GBM+Treatment+Response) and CPTAC‐GBM (https://wiki.cancerimagingarchive.net/pages/viewpage.action?pageId=30671232). The inclusion criteria were as follows: (a) pathological confirmation of newly‐diagnosed GBM (Grade IV) or LGG (Grade II‐III) according to the 2016 CNS WHO^10^; (b) obtained pre‐treatment, high‐resolution T2‐weighted images. A total of 396 GBM and 190 LGG (115 astrocytoma and 75 oligodendroglioma) patients from our center and TCIA repository were finally included for further imaging processing (Figure 2). Among all GBM patients, molecular features were available in TCGA‐GBM cohort and our center. To further investigate the spatial distribution of GBM, 107 patients diagnosed as IDH‐wildtype GBM were chosen as an independent subgroup. The baseline characteristics of the selected GBM and LGG patients were summarized in Table 1 and detailed information of IDH‐wildtype GBM, astrocytoma and oligodendroglioma could be found in Table S1. In addition, 108 GBM patients with full clinical information, including age, sex, Karnofsky Performance Status (KPS) and accurate survival time, were used to build a GBM prognosis prediction model.

**TABLE 1:** General information of selected patients

	GBM	LGG	*P* value
Total number	396 (100)	190 (100)	
Gender			
Male	117 (29.5)	86 (45.3)	.172^a^
Female	87 (22.0)	85 (44.7)	
Not available	192 (48.5)	19 (10.0)	
Age (y)			
≥50	266 (67.2)	64 (33.7)	<.001^a^
<50	63 (15.9)	109 (57.4)	
Not available	67 (16.9)	17 (8.9)	
Mean ± SD	60.02 ± 13.17	43.29 ± 14.16	<.001^b^
KPS			
≥90	25 (6.3)	54 (28.4)	<.001^a^
<90	89 (22.5)	25 (13.2)	
Not available	282 (71.2)	111 (58.4)	
Mean ± SD	81.51 ± 14.12	86.86 ± 12.09	.023^b^
Survival (y)			
≥1	175 (44.2)	93 (48.9)	<.001^a^
<1	164 (41.4)	16 (8.4)	
Not available	57 (14.4)	81 (42.6)	
Mean ± SD	14.35 ± 13.39	40.94 ± 35.27	<.001^b^
Tumor volume (cm^3^)			
<40	297 (75.0)	123 (64.7)	.010^a^
≥40	99 (25.0)	67 (35.3)	
Mean ± SD	29.62 ± 25.25	44.69 ± 54.18	.027^b^

*Note*: Data are shown as the number of patients with percentages in parentheses or Mean ± SD. *P* value calculations exclude the category “Not available.”

^a^ Results of Chi‐square test.

^b^ Results of *t* test.

### 2.2 Tumor segmentation, coregistration and normalization

Preoperative T2‐weighted images, which could reflect the extent of tumor invasion,^17^ were analyzed as a reference sequence. The parameters of T2 images acquired from our institution were as following: flip angle 8°, matrix 256 × 256 and slice thickness 5 mm. For the images from TCIA, most had modalities obtained with different acquisition parameters and those whose resolution was low were excluded from this research. Two experienced neuroradiologists manually delineated lesion margins. Those tumor boundaries, whose discrepancy was more than 5%, were re‐evaluated. The lesion tissue was encoded as a 1/0‐mask (1 tumor, 0 background) and nonlinearly registered to Montreal Neurological Institute (MNI) space (Montreal, Quebec, Canada). The glioma frequency maps were constructed by dividing the sum of weighted glioma lesion masks by the total number of patients from HMU Cancer Hospital and TCIA in each cohort on a voxelwise basis.

### 2.3 Voxel‐based lesion‐symptom mapping analysis

To quantify the potential spatial distribution differences between GBM and LGG, voxel‐based lesion‐symptom mapping (VLSM) analysis was performed using the VLSM toolbox in MATLAB. Voxels with strong correlations with GBM or LGG histological subtype were identified after controlling for the effects of age, sex and tumor volume. Only those voxels with *t* values higher than those from more than 95% of permutations (*n* = 500) were finally preserved.^18^ Using this method, we also identified a specific region associated with shorter OS in GBM. GBM‐associated regions and GBM short‐OS‐associated regions were defined as region of interest (ROI)1 and ROI2, respectively.

### 2.4 Transcriptomic correlation with glioma frequency map

In characterizing the intrinsic human brain gene expression profile, we obtained regional microarray expression data of six postmortem brains from the Allen Human Brain Atlas (AHBA; https://human.brain-map.org/static/download). Only samples from the left hemisphere were used in consideration of reliability. The data were processed as previous research and mapped to the same Brainnetome atlas as above. All processing was conducted with the abagen toolbox (https://github.com/netneurolab/abagen) following previous work.^19^

In brief, the probes were reannotated and those with the highest differential stability were selected as the representatives of each gene. Next, samples were assigned to the nearest Brainnetome region. Gene expression values were normalized across samples and genes for each donor to reduce the potential intersubject variance. The expression values of samples specified to the corresponding region were averaged for each individual, followed by averaging across donors. Finally, an expression profile of 15 655 genes across 246 regions was generated. Partial least square regression (PLS) was leveraged to disclose the relationship between the gene expression profile and tumor distribution. The SIMPLS algorithm was used to maximize the covariance between the linear combination of gene expression (*X*) and tumor distribution (*Y*). The square rooted tumor frequency was used to reduce the skewness. Singular value decomposition was conducted on $Y^{\prime }X$:

(1) $${\left(Y^{\prime }X\right)}^{\prime }=\mathrm{US}V^{\prime }$$

where *U* and *V* are matrices of left and right singular vectors, together with a diagonal matrix *S* of singular values representing the covariance of singular vectors. We further assessed the significance of the association by a spatial permutation test. Especially, the spin test was applied to each singular value in *S* by applying PLS to the original expression matrix and spun tumor distribution matrix. The *P‐value* was calculated as the proportion of singular values from a null distribution that was greater than the actual singular value, which represented the probability of random association because of pure spatial autocorrelation.

Following up the PLS analyses on tumor distribution, we used columns in *U* to rank genes in descending order by their loadings in each PLS analysis, which were further tested for the distribution in GO biological processes with generally applicable gene‐set enrichment (GAGE). A threshold of false discovery rate (FDR) corrected *P* < .05 was set for both.

### 2.5 Neurotransmitter correlation with Glioma frequency map

The JuSpace toolbox (https://github.com/juryxy/JuSpace) was employed to conduct spatial correlation of glioma frequency with nuclear imaging derived neurotransmitter receptor distribution data.^20^ The Juspace toolbox provides normative brain maps of neurotransmitter receptors, including gamma‐aminobutyric acid type A (GABAa),^21^ serotonin 5‐hydroxytryptamine receptor subtype 1a (5‐HT1a),^22^ serotonin transporter (SERT),^22^ dopamine D2 (D2),^23^ dopamine transporter (DAT),^21^ and noradrenaline transporter (NAT).^24^ Spearman's correlation was estimated between the glioma frequency map and each neurotransmitter receptor map separately.

### 2.6 Differential gene expression analysis of tumors located in predilection and nonpredilection sites

In the glioma frequency map, a higher voxel value indicated a higher tumor occurrence rate in this corresponding voxel. Voxels with the top 10% maximum value were extracted as a predilection site mask, representing the brain regions in which GBM or LGG occurred preferably. For each patient, we calculated the overlapping tumor volume and proportion with a predilection site mask. All patients from our center and TCIA were arranged according to their overlapping volume or proportion value in descending order. Those who ranked in the top 20% or bottom 20% of overlapping volume and proportion were selected. RNA‐seq profiles for glioma tissues are available with some of these patients from the TCIA dataset. Specifically, tumor RNA‐seq data from 20 patients in the GBM group (top 20%, n = 12; bottom 20%, n = 8) and 25 patients in the LGG group (top 20%, n = 12; bottom 20%, n = 13) were used to screen differentially expressed genes (DEGs) using R package DESeq2 (|log_2_ FC| > 2 and *P*_adj_ < .05). To identify the biological functions of these spatial distribution‐related DEGs, we used the R package “clusterProfiler” to conduct GO enrichment and Kyoto Encyclopedia of Genes and Genomes (KEGG) pathway analyses (*P*_adj_ < .05). Gene set enrichment analysis (GSEA) which can detect significant differences in various biological functions, was carried out by the R package “clusterProfiler” (*P*_adj_ < .05). We then applied the Search Tool for the Retrieval of Interacting (STRING) (https://string-db.org/) to evaluate the Protein‐Protein Interaction (PPI) network (confidence score ≥0.4). The top 10 hub genes were identified by the degree method (see Table S2 for details).

### 2.7 Survival analysis

To explore the correlation between DEG expression and survival time, we downloaded RNA‐seq and prognosis data from the GBM (n = 173) and LGG (n = 529) cohorts in The Cancer Genome Atlas (TCGA) data portal (https://tcga-data.nci.nih.gov/tcga/).^25^ In each cohort, patients were divided into upregulated and downregulated groups by the median gene expression value. Furthermore, patients in our cohort were grouped by quartiles of overlapping volume/proportion and distance to ROI1/ROI2/SVZ. Survival analysis between two groups was generated by Kaplan‐Meier log‐rank test.

### 2.8 Construction of a prognostic model based on hub genes

We used the least absolute shrinkage and selection operator (LASSO) and multivariate Cox regression analyses to assess the relationship between prognostic hub genes expressions and overall survival in TCGA by R package “glmnet.” The most valuable prognostic hub genes selected from LASSO analysis were evaluated by the Cox regression risk model. The risk score of each patient was calculated based on gene expression levels and regression coefficient as follows: risk score = ∑ (coefficient × gene expression). The median value of risk score was used to divide the TCGA GBM patients into low‐ and high‐risk subgroups. Kaplan‐Meier curves and log‐rank tests methods were applied to compare the overall survival between the two groups. Finally, the receiver operating characteristic (ROC) curve was calculated to evaluate the prognostic ability of the constructed model at 1‐year and 3‐year endpoints with the R package “timeROC.”

### 2.9 Quantification of tumor location features

To characterize tumor location features, we calculated the mean distance of individual tumors to the above derived sets of areas (ROI1 and ROI2). Specifically, we generated two masks at the mass center of ROI1 and ROI2, followed by calculating the mean distance of each individual tumor to the center masks using the distance map tool in FSL. Moreover, we also calculated the distance of individual tumors to the SVZ. The SVZ areas were defined by constructing a lateral ventricular mask in MNI space using the FSL segmentation algorithm BIANCA with further manual refining. As previously described, we then calculated the closest distance of each voxel in the brain to this ventricular mask using a distance map in FSL, resulting in a concentric periventricular distance map.

### 2.10 Prediction of GBM 1‐year survival status

In exploring the contribution of the tumor's anatomical position to the survival state at 1 year after diagnosis, we concentrated on the areas of high occurrence in GBM (ie, ROI1 and ROI2 derived above), which were used to predict survival time. Specifically, we calculated the overlapping volume and proportion of individual tumors with these areas, as well as the mean Euclidean distance of individual tumors to these areas. All these metrics, together with tumor volume, age, sex and KPS, were included in support vector machine (SVM) models. Two SVM models using linear or radial kernels were constructed with a caret package in R. A grid search parameter tuning strategy was used. The relative importance of the features for the overall prediction was assessed by a conditional feature importance score.

To validate the performance of this model, another 20 newly diagnosed GBM patients treated at our neurosurgery department in 2019 were selected (baseline characteristics are described in Table S3). A total of 108 patients from the GBM cohort (Dataset 1) and an additional independent cohort of 20 GBM patients (Dataset 2), whose clinical information (age, sex, KPS and accurate survival time) was complete, were used for prediction analyses. We leveraged a repeated stratified random sampling plus cross‐validation scheme for the training and testing of the prediction models. Specifically, for each time, 80% of patients from Dataset 1 and 80% of those from Dataset 2 were stratified random sampled (based on survival status) and pooled as the training dataset to minimize the potential distribution differences of the two datasets and class labels, while the resting out‐of‐bag patients were pooled to test the model generalizability. At each sampling time, 10‐fold cross‐validation was used to help choose the optimal model parameter, and the ROC metric was applied to evaluate the performance. This training‐testing process was repeated 100 times to evaluate the robustness of these GBM prediction models against particular ways of splitting data. The final performances of the models were reported as the mean and SD of the area under the ROC curve (AUC).

## 3 RESULTS

### 3.1 Differences in spatial distribution between GBM and LGG

**FIGURE 3:**
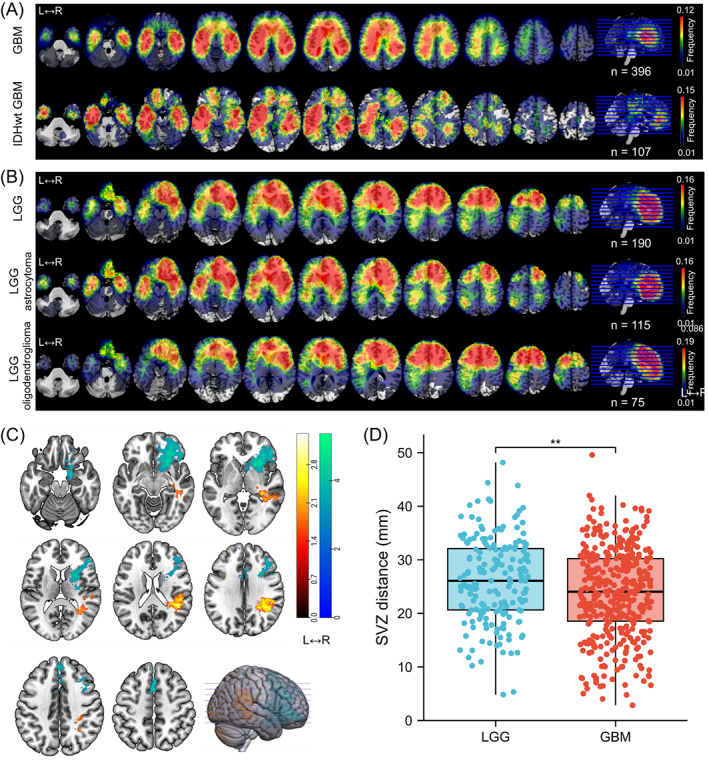
Differences in spatial distribution between GBM and LGG. (A,B) Frequency distribution of GBM and LGG, respectively. Three hundred ninety‐six GBM patients and 190 LGG patients from HMU Cancer Hospital and TCIA were included in the further analysis. The frequency maps of IDH‐wildtype GBM (n = 107), astrocytoma (n = 115) and oligodendroglioma (n = 75) subgroups were shown independently. The color bar illustrates the frequency proportion of different cohorts, from blue (1 case) to red. Images are displayed in neurologic convention orientation. (C) VLSM‐identified clusters associated with GBM or LGG. Only significant voxels are rendered in red (GBM) or blue (LGG) based on a permutation test (n = 500, *P* < .01). A larger t value indicates a stronger correlation. (D) Tumor average distance to the SVZ. Wilcoxon rank‐sum test, *P* = .002

A total of 190 patients with LGG (115 astrocytoma and 75 oligodendroglioma) and 396 patients with GBM (including 107 identified IDH‐wildtype GBM) from HMU Cancer Hospital and TCIA repository who met the inclusion criteria were enrolled in the study (Figure 2). The clinical and radiologic characteristics of LGG and GBM patients are detailed in Tables 1 and S1. Similar to previous research, we observed that gliomas mainly occurred in the frontal lobe, temporal lobe, insula and SVZ (Figure 3A,B). Furthermore, astrocytoma and oligodendroglioma were more likely to be distributed in the anterior regions, while there was more frequent involvement of the posterior regions around the SVZ in IDH‐wildtype GBM and whole GBM cohorts. To further compare the differences, we applied VLSM analysis to identify specific regions that were correlated with GBM or LGG. The clusters correlated with GBM were predominantly located in the right temporal lobe, postcentral gyrus, parietal operculum cortex and angular gyrus, whereas clusters correlated with LGG were located in the right frontal lobe, caudate, putamen and insular cortex (Figure 3C). Given that the SVZ is the largest source for stem and progenitor cells that initiate gliomagenesis, we calculated the distance from each individual's tumor to the lateral ventricle border and found that on average, LGG was located further from the SVZ than GBM (23.77 ± 8.36 vs 26.25 ± 7.82 mm, *P* = .002) (Figure 3D).

### 3.2 Intrinsic transcriptomic correlates of glioma spatial distribution

**FIGURE 4:**
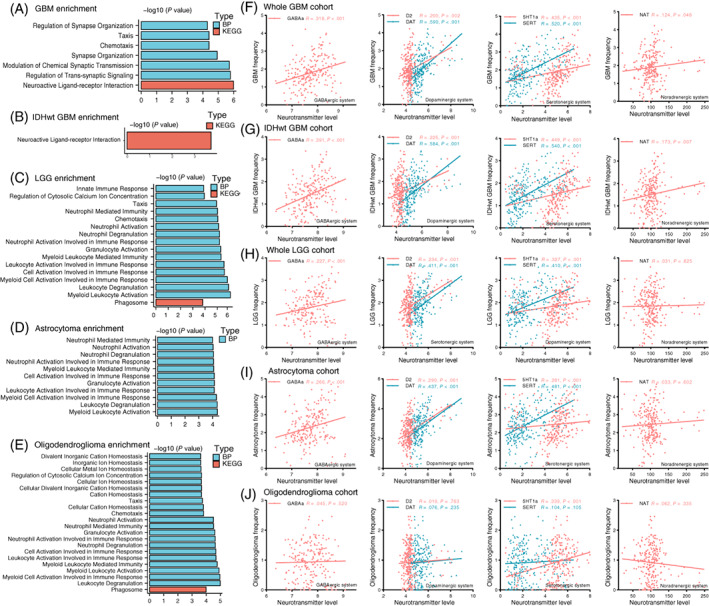
Transcriptomic and neurotransmitter correlates of glioma spatial distribution. (A‐E) GO and KEGG functional enrichment results of tumor location associated genes in whole GBM, IDH‐wildtype GBM, whole LGG, astrocytoma and oligodendroglioma, respectively. FDR corrected *P* < .05. (F‐J) Spearman correlation analysis between tumor frequency and neurotransmitter system level in different brain areas. All patients came from a combined collection of HMU Cancer Hospital and TCIA. (A,F) Whole GBM; (B,G) IDH‐wildtype GBM; (C,H) Whole LGG; (D,I) Astrocytoma; (E,J) Oligodendroglioma. 5‐HT1a, serotonin 5‐hydroxytryptamine receptor subtype 1a; D2, dopamine D2; DAT, dopamine transporter; GABAa, gamma‐aminobutyric acid type A; NAT, noradrenaline transporter; SERT, serotonin transporter

Next, to further investigate the factors that might affect glioma location predilection, PLS was conducted to disclose the relationship between the expression of 15 655 genes across 246 regions and tumor distribution. Ultimately, all genes were entered into functional enrichment analysis. Genes corresponding to GBM spatial location were linked to neuroactive ligand‐receptor interactions and biological processes such as regulation of synapse organization, synapse organization, modulation of chemical synaptic transmission and neuroactive ligand‐receptor interactions (Figure 4A). In IDH‐wildtype GBM, the enrichment of neuroactive ligand‐receptor interaction was also observed (Figure 4B). In contrast, genes that correlated with LGG distribution (Figure 4C), including astrocytoma (Figure 4D) and oligodendroglioma (Figure 4E), were mostly associated with immunity response‐related biological processes, especially neutrophil and leukocyte‐mediated immune response.

### 3.3 Spatial correlation between neurotransmitter system level and glioma frequency

To validate neuron and glioma synaptic interactions were involved in GBM location predilection, we analyzed the spatial correlation of tumor distribution with positron emission tomography‐derived average neurotransmitter maps, covering GABAergic (GABAa), dopaminergic (D2, DAT), serotonergic (5HT1a, SERT) and noradrenergic (NAT) systems. As shown in Figure 4F,G, tumor frequency in GBM and IDH‐wildtype GBM was significantly positively correlated with all of the six neurotransmitter levels. In contrast, LGG frequency was only significantly correlated with GABAergic, dopaminergic and serotonergic maps and the Spearman correlation coefficients of LGG were smaller than those in GBM (Figure 4H). When comparing different LGG subgroups, we found that tumor frequency in astrocytoma was significantly correlated with GABAergic, dopaminergic and serotonergic maps (Figure 4I), while only a positive correlation in 5HT1a was observed in oligodendroglioma (Figure 4J).

### 3.4 Transcriptional signatures of glioma located at predilection sites

**FIGURE 5:**
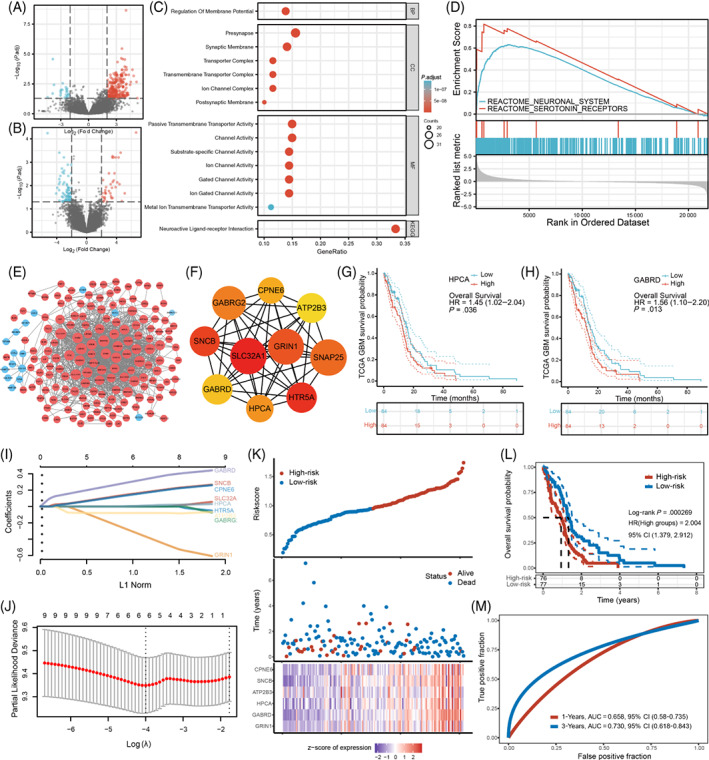
Transcriptomic analysis of tumors located in predilection and nonpredilection sites. (A,B) Volcano plots of DEGs in GBM and LGG. Significant DEGs are highlighted (|log_2_ FC| >2 and adjusted *P value* <.05). Red dots indicate upregulated DEGs and blue dots indicate downregulated DEGs. (C) GO and KEGG functional enrichment results of DEGs in GBM. Terms with adjusted *P values* <.001 and counts ≥20 are shown in the figure. (D) Results of GSEA in GBM. NES: normalized enrichment score. (E) Integrated PPI network for DEGs in GBM. Nodal color and size refer to the log FC of gene expression and the number of interacting proteins, respectively. Blue: upregulation; Red: downregulation. (F) Interaction networks of the top 10 hub genes. The color depth indicates the ranking of hub genes. (G,H) OS analysis of hub genes HPCA and GARBD in the TCGA database. (I,J) LASSO Cox regression was used to build a prognostic model based on hub genes. (K) The distributions of the risk score and survival status in the TCGA‐GBM cohort. A heat map indicated the hub gene expression profiles of the two groups. (L) Kaplan‐Meier curves for the OS of the TCGA‐GBM patients in the low‐risk group and high‐risk group (*P* < .001). (M) The AUC of time‐dependent ROC curves demonstrated the predictive efficiency of the risk score. Transcriptomic analyses were conducted using the data from HMU Cancer Hospital and TCIA (A‐F). Survival analyses of the hub genes were performed in TCGA database (G‐M)

Next, we sought to assess whether gliomas located at predilection sites would exhibit biological behaviors distinct from those located at nonpredilection sites, as reflected by differential gene expression. We selected patients from our center and TCIA whose glioma was located at the predilection or nonpredilection regions and conducted transcriptomic analysis (see Section 2 for details). In GBM, we identified a total of 272 DEGs, including 248 upregulated genes and 24 downregulated genes in tumor tissues from high‐frequency regions (Figure 5A). In LGG, a total of 111 DEGs were identified for tumors located at the predilection site, of which 37 were upregulated and 74 were downregulated (Figure 5B). Fourteen significant terms in GO analysis revealed that most DEGs were enriched in synapse, transporter activity and channel activity‐related functions (adjusted *P* < .001, counts ≥20) (Figure 5C). In KEGG analysis, “neuroactive ligand‐receptor interaction” was the only significantly upregulated pathway. In GSEA, the most significantly enriched signaling pathways were “neuronal system” and “serotonin receptors” (Figure 5D). In contrast, there were no significant GO, KEGG or GSEA results in LGG.

To investigate the interactions between DEGs in GBM, we constructed a PPI network consisting of 173 nodes and 1269 edges (Figure 5E). Among the top 10 upregulated hub genes (Figure 5F), six genes (SLC32A1, HTR5A, GRIN1, SNAP25, GABRG2, GABRD) are involved in neurotransmitter release, reuptake and degradation; two genes (HPCA, ATP2B3) play a critical role in calcium ion channels and intracellular calcium homeostasis; and two genes (SNCB, CPNE6) function in neuroplasticity (see Table S2 for details). To test whether these hub genes would affect prognosis of GBM patients, we conducted Kaplan‐Meier survival analysis in the TCGA database, and revealed that patients with an increased HPCA or GARBD expression had poorer long‐term survival (Figure 5G,H). No significant results were found for the other hub genes (Table S2).

Furthermore, we constructed a prognostic model based on hub gene expression signature. LASSO regression analysis identified six valuable hub genes (CPNE6, SNCB, ATP2B3, HPCA, GABRD and GRIN1) that were significant predictors of survival time in TCGA‐GBM cohort (Figure 5I,J). These hub genes were further included in Cox regression analysis to construct a prognostic risk model with the following formula: risk score = 0.1507 × exp SNCB − 0.2949 × exp GRIN1+ 0.0044 × exp HPCA +0.1415 × exp CPNE6 + 0.2997 × exp GABRD − 0.081 × exp ATP2B3. According to the median risk score, GBM patients in TCGA database were classified into the low‐risk and high‐risk subgroups (Figure 5K). Patients in the high‐risk group exhibited shorter survival time than those in the low‐risk group (*P* < .001, Figure 5L). ROC analysis revealed that the AUC was 0.658 for 1‐year survival and 0.730 for 3‐year survival (Figure 5M), indicating that they had prognostic value in GBM.

### 3.5 Features affecting the overall survival of GBM patients

**FIGURE 6:**
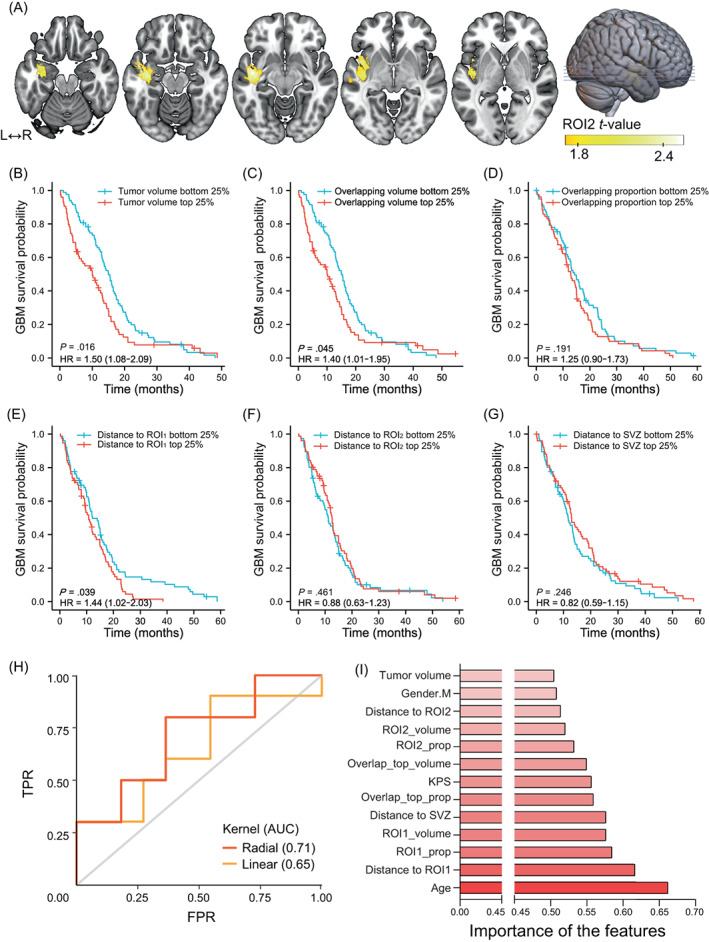
Imaging topological features and GBM survival time. (A) Anatomical correlates of shorter OS in GBM as identified by VLSM. Only significant voxels are rendered in yellow. (B‐G) Kaplan‐Meier survival curve analysis between the highest quartile group and the lowest quartile group of six imaging topological features: tumor volume, overlapping volume and proportion with top 10% high‐frequency regions, overlapping proportion with top 10% high‐frequency regions, distance to ROI1, distance to ROI2 and distance to SVZ. (H) The AUC for the SVM prediction model. (I) The importance of selected features included in the SVM classifier. All GBM patients came from the combined collection of HMU Cancer Hospital and TCIA

Next, we identified short‐OS‐associated regions by VLSM. Tumors occurring in the left insular cortex, parahippocampal gyrus, superior temporal gyrus, middle temporal gyrus, Heschl's gyrus and central opercular cortex tended to lead to poorer survival (Figure 6A). GBM‐associated regions (Figure 3C) and GBM short‐OS‐associated regions (Figure 6A) were defined as ROI1 and ROI2, respectively. To test whether tumor size or location could affect the prognosis of GBM, GBM patients from HMU Cancer Hospital and TCIA were grouped according to tumor volume, overlapping volume and proportion with the top 10% frequency mask and distance to ROI1, ROI2 and SVZ. The survival curves of patients from the highest quartile (top 25%) group and lowest quartile (bottom 25%) group were compared by the Kaplan‐Meier method. Patients with a larger tumor volume showed significantly less OS than those with smaller volume (*P* = .016, Figure 6B). A similar result was found in the overlapping volume (*P* = .045, Figure 6C) but not in the overlapping proportion (*P* = .191, Figure 6D). However, no meaningful differences were found in LGG (Figure S1). Among the three distance metrics (distance to ROI1/ROI2/SVZ), only distance to ROI1 was statistically significant, with a closer distance to ROI1 indicating better survival in GBM (*P* = .039, Figure 6E‐G). In LGG, the total number of patients with accurate survival time was not enough for further analysis.

### 3.6 Construction and validation of the GBM prognosis prediction model

In an attempt to confirm the potential contributions of tumor topographic features to the prediction of the 1‐year survival status of GBM patients, we constructed linear and nonlinear (radial) kernel‐based SVM models using the quantified tumor metrics above, together with age, sex and KPS. In 100 repeated random samplings, the radial kernel (AUC = 0.71 ± 0.09) could better predict the survival status than the linear (AUC = 0.65 ± 0.13) model (Figure 6H). Importantly, among the potential predictors, the conditional feature importance score was higher for age, KPS, distance to ROI1, ROI1 overlapping proportion, ROI1 overlapping volume and distance to SVZ (Figure 6I).

## 4 DISCUSSION

In the current study, we constructed a population‐based frequency map for 396 patients with GBM and 190 patients with LGG, including 115 astrocytoma and 75 oligodendroglioma. We confirmed that the spatial distribution pattern of GBM differed from that of LGG. In addition, astrocytoma and oligodendroglioma displayed similar spatial distribution. To investigate the underlying mechanism, we first analyzed the correlation between whole brain gene expression and glioma frequency. Next, we explored the transcriptomic characteristics of brain regions frequented by GBM and LGG. We found that GBMs were more likely to occur at areas enriched with synaptic signaling‐related genes, whereas astrocytoma and oligodendroglioma location was associated with immune and inflammation response. Compared to glioma located in nonpredilection regions, GBMs located at predilection sites were demonstrated to show elevated expression of gene sets enriched in synapse, neurotransmitter and channel activity‐related pathways. Moreover, the hub genes, which might affect tumor site predilection, were strongly correlated with survival time. Finally, we revealed that topographical features of GBM, such as tumor size and location, were associated with the OS of patients, and could successfully predict prognosis for patients with GBM using a nonlinear SVM model.

### 4.1 Spatial distribution of gliomas

Tumorigenesis may have an impact on glioma spatial distribution patterns across the brain.^26^ It has been widely suggested that NSCs are involved in the origin of gliomas, and OPCs are the major cells of origin of LGG.^12^ In support of this notion, we observed that both GBM and LGG exhibited a high probability of tumor location along the lateral wall of the lateral ventricles and in the hippocampus, which are the largest germinal regions of NSCs in the adult brain.^27^ Interestingly, we observed that GBM exhibited a higher frequency in the posterior part of the SVZ, while LGG had a relatively higher incidence in the frontal lobe. These differences, which could not be explained by tumor origin, might be a result of evolutionary adaptation of the tumor to the TME.

### 4.2 Inflammation and immune TME in glioma maintenance

We noticed distinct transcriptional correlates for LGG and GBM. Specifically, both low‐grade astrocytoma and oligodendroglioma tended to occur in regions enriched with the neutrophil immune response (Figure 4C‐E), which is consistent with prior studies reporting that there is more neutrophil infiltration in the glioma permissive environment.^28^ This might be a result of adaptation in response to immunity during the lengthy disease course. In contrast, for GBM, no immune‐related pathways were significant (Figure 4A,B), which may reflect their limited antitumor immune response. Although the brain has long been considered a site of immune privilege, recent studies suggest that immune cells play an active role in diverse CNS diseases.^29^ It has been proven that cancerous cells and tumor‐associated neutrophils/macrophages could trigger inflammatory responses and this chronic inflammatory TME increases the survival capacity of glioma cells.^30^, ^31^ Various inflammatory mediators, such as cytokines, growth factors and chemokines, not only promote glioma cell proliferation and invasion but also assist in the recruitment of immune cells of TME, like tumor‐associated neutrophils, that in return contribute to glioma maintenance and progression.^32^ This may explain why LGG tended to be located in areas with strong inflammation and immune signature (Figure 4C‐E).

### 4.3 Neuron and glioma interactions

One important finding of the present study is that neuron and glioma interactions, especially neurotransmitters (glutamate, GABA, 5‐hydroxytryptamine) and calcium ion channel‐related pathways are likely to be involved in GBM site predilection (Figures 4A,D and 5C). In line with our observations, synapse‐associated genes, including the postsynaptic structure and glutamate receptor, have been found to be overexpressed in several malignant glioma subpopulations.^33^ Recent advances suggested that electrochemical communications between neurons, NSCs, OPCs and glioma cells are essential to tumor growth and progression.^34^ In glial cells, NSCs and glioblastoma stem cells (GSCs), the glutamatergic system triggers Ca^2+^ influx and finally elevates intracellular Ca^2+^ level. As a ubiquitous second messenger, a high concentration of intracellular Ca^2+^ promotes cell duplication and migration and induces transformation to glioma cells.^35^ Meanwhile, glioma cells release more glutamate to the TME in response to high Ca^2+^ concentration stimulation.^36^ On the one hand, this excitotoxicity negatively regulates adjacent neurons; on the other hand, excessive glutamatergic and calcium ion signaling stimulate the progression of glioma.^37^ Furthermore, GABA, a main inhibitory neurotransmitter in the mammalian brain, also participates in this interaction network. Although activation of the GABA‐A receptor can depolarize NSCs and glioma cells, different GABA‐A channel subtypes have a distinct impact on glioma proliferation and clinical outcome.^38^ Building on this evidence, our results indicate that the activation of complex communications between GBM cells and other nonmalignant cells, involving a variety of neurotransmitters, ion channels and postsynaptic receptors, also contributes to determining the tumor location predilection. We found that GBM distribution had a stronger spatial correlation with GABAergic, dopaminergic, serotonergic and noradrenergic systems than astrocytoma and oligodendroglioma (Figure 4F‐J). Therefore, we anticipate that novel therapies targeting neuron‐glioma interactions may hold promise to benefit GBM patients, especially those with tumors localized at high‐risk regions.

### 4.4 Prognosis prediction of gliomas

Gliomas are characterized by high complexity and heterogeneity. Therefore, individual prediction of survival time is of great clinical value for patients with glioma. Conventional prediction models are usually based on clinical and molecular information.^39^ In recent studies, gene signature, immune infiltration or other feature sets were also incorporated when building the prediction model.^40^ However, all these predictors are limited by the availability of glioma samples. Only after tumor resection or biopsy can they perform predictive analysis. To overcome this difficulty, preoperative imaging‐based methods are optional choices. Some studies extract shape or volumetric features to characterize the distinct phenotypes of gliomas (ie, active tumor, necrotic regions and peritumoral edema).^41^ Other researchers extract radiographic characteristics through machine learning or deep learning, which are more objective and comprehensive.^42^ However, these strategies require multiparametric imaging data. Compared to these prior models, our preoperative method, relying only on GBM anatomic location features and basic patient information (age, sex and KPS), is noninvasive, convenient, inexpensive and easy to operate. Notably, our results demonstrated that using these simple metrics could achieve an accuracy comparable to previous literature.^43^, ^44^ To our knowledge, this is the first study that takes advantage of tumor location characteristics to predict the prognosis of patients with glioma. We believe that a combination of these characteristics with other features, such as histopathology and molecular alterations, may also contribute to improving the predictive accuracy.

### 4.5 Limitations

First, to maximize the sample size and extend the generalizability of our findings, we collected imaging data from multiple centers. The diverse scanners and parameters might have some influence on data preprocessing and frequency mapping. Second, some key molecular alterations, such as IDH mutation and O6‐methylguanine‐DNA methyltransferase (MGMT) promoter methylation, have been shown to affect glioma location predilection.^45^ Considering the intertumoral heterogeneity of GBM, future studies are warranted to divide GBMs into molecular subgroups and discuss them separately. However, the molecular data of the patients enrolled in our study were incomplete. Among all the 396 GBM patients, only the patients from TCGA‐GBM and our center had confirmed molecular information. A total of 107 patients were diagnosed as IDH‐wildtype GBM and we chose them as an independent cohort to validate our findings in the whole GBM cohort. IDH‐wildtype GBM also had a higher frequency in the temporal lobes and posterior part of the SVZ (Figure 3A). Gene enrichment and spatial correlation analyses indicated that neuron and glioma interactions were involved in IDH‐wildtype GBM location predilection (Figure 4B,G). Another limitation is that the total number of LGG patients with full survival information was not sufficient to establish an outcome prediction model. Large sample studies focusing on LGG localization features and prognosis are needed.

## 5 CONCLUSION

An in‐depth understanding of glioma localization patterns unravels the mystery of distinct pathophysiological behaviors between GBM and LGG. The spatial distribution of GBM is not random and differs significantly from that of LGG. We revealed that both the pretumor microenvironment and tumor tissues from high‐frequency regions were enriched with genes associated with synapse and neurotransmitter‐related signaling pathways in GBM, which brought a new perspective to interpret glioma location predilection, gliomagenesis and neurogliomal synapse. We have generated a multitude of prognosis‐related spatial distribution signatures, which herald the promise for the potential application of using tumor location features to predict outcome and treatment responses to individualized cancer therapies.

## AUTHOR CONTRIBUTIONS

**Hongbo Bao:** Original conception of this research; data acquisition; technical and experimental work; drafted the article and conducted statistical analyses. **Peng Ren:** Original conception of this research; technical and experimental work; critically revised the article; drafted the article and conducted statistical analyses. **Liye Yi:** Original conception of this research; technical and experimental work. **Zhonghua Lv:** Technical and experimental work. **Wencai Ding:** Technical and experimental work. **Chenlong Li:** Technical and experimental work. **Siyang Li:** Technical and experimental work. **Zhipeng Li:** Technical and experimental work. **Xue Yang:** Data acquisition. **Xia Liang:** Original conception of this research; critically revised the article. **Peng Liang:** Original conception of this research. The work reported in the article has been performed by the authors, unless clearly specified in the text.

## FUNDING INFORMATION

X.L was supported by the National Natural Science Foundation of China (Nos. 82072000 and 81671769), the Fundamental Research Funds for the Central Universities of China (No. HIT. NSRIF. 2020042) and the Natural Science Foundation of Heilongjiang Province, China (Grant No. LH2019H001). L.Y was supported by Heilongjiang Postdoctoral Scientific Research Developmental Fund (LBH‐Q21111). Z.L was supported by Haiyan Foundation (JJMS2021‐29). H.B. was supported by a grant from Harbin Eagle Project (2020CYJBNS0520).

## CONFLICT OF INTEREST

The authors report no competing interests.

## ETHICS STATEMENT

All glioma patients from HMU Cancer Hospital were informed and ethical approval for the current research was obtained through the Ethics Committee of Harbin Medical University (KY2021‐42).

## Supporting information


**Figure S1.** Imaging topological features and LGG survival time.
**Table S1.** Baseline characteristics of glioma subgroups.
**Table S2.** Top 10 hub genes in the interaction network.
**Table S3.** Baseline characteristics of the selected GBM patients.

## Data Availability

Data sources and handling of publicly available data are described in the Materials and Methods. The MRI data from HMU Cancer Hospital can be downloaded from public repository SYNAPSE (https://www.synapse.org/#!Synapse:syn34152292/files/). Further information is available from the corresponding author upon request.
