# Supplementary material for: New insights into glioma frequency maps: From genetic and transcriptomic correlate to survival prediction
Source: Int J Cancer. 2022 Nov 24;152(5):998–1012. doi: 10.1002/ijc.34336 (PMC10100131; doi:10.1002/ijc.34336)
Supplement: Supplementary file 1 — Figure S1. Imaging topological features and LGG survival time. Table S1. Baseline characteristics of glioma subgroups. Table S2. Top 10 hub genes in the interaction network. Table S3. Baseline characteristics of the selected GBM patients. [file IJC-152-998-s001.pdf]

New Insights into Glioma Frequency Maps: From Genetic and Transcriptomic Correlate to Survival Prediction

Hongbo Bao, Peng Ren, Liye Yi, Zhonghua Lv, Wencai Ding, Chenlong Li, Siyang Li, Zhipeng Li, Xue Yang, Xia Liang, Peng Liang

Table of Contents:

**Supplementary figure**	Imaging topological features and LGG survival time
**Supplementary Table 1**	Baseline characteristics of glioma subgroups
**Supplementary Table 2**	Top 10 hub genes in the interaction network
**Supplementary Table 3**	Baseline characteristics of the selected GBM patients

Supplementary figure

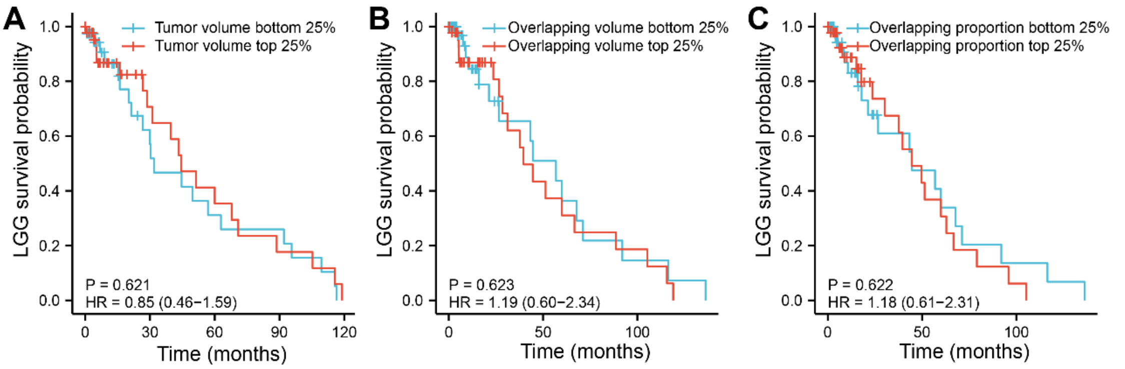

Imaging topological features and LGG survival time. (A-C) Kaplan–Meier survival curves analysis between the highest quartile group and the lowest quartile group of three imaging topological features: tumor volume, overlapping volume with the top 10% highest frequency regions, overlapping proportion with the top 10% highest frequency regions.

Supplementary Table 1. Baseline characteristics of glioma subgroups

	IDHwt GBM	Astrocytoma	Oligodendroglioma	*P* value
**Total Number**	107(100)	115 (100)	75 (100)	
**Gender**				
Male	52 (48.6)	49 (42.6)	37 (49.3)	0.935[a]
Female	55 (51.4)	50 (43.5)	35 (46.7)	
Not available	0 9 (0)	16 (13.9)	3 (4.0)	
**Age (y)**				
≥ 50	74 (69.2)	34 (29.6)	30 (40.0)	<0.001[a]
<50	33 (30.8)	66 (57.4)	43 (57.3)	
Not available	0 (0)	15 (13.0)	2 (2.7)	
Median (IQR)	61 (47, 66)	40 (31, 54)	44 (33, 54)	<0.001[b]
**KPS**				
≥ 90	21 (19.6)	33 (28.7)	21 (28.0)	<0.001[a]
< 90	52 (48.6)	17 (14.8)	8 (10.7)	
Not available	34 (31.8)	65 (56.5)	46 (61.3)	
Median (IQR)	80 (80, 97.5)	90 (80, 90)	90 (82.5, 90)	0.034[b]
**Tumor Volume (cm^3)**				
<40	75 (70.1)	77 (67.0)	46 (61.3)	0.465[a]
≥40	32 (29.9)	38 (33.0)	29 (38.7)	
Median (IQR)	27.3 (14.9, 41.1)	26.4 (12.7, 53.1)	26.6 (11.0, 59.0)	0.948[b]

Data are shown as the number of patients with percentages in parentheses or Mean ± SD.

[a]Results of chi-square test.

[b]Results of Kruskal-Wallis test.

P value calculations exclude the category 'Not available'.

Supplementary Table 2. Top 10 hub genes in the interaction network

Rank	Name	Score	OS *P*-value	Full name
1	SLC32A1	9.98E+12	0.503	solute carrier family 32 (GABA vesicular transporter), member 1
2	HTR5A	9.93E+12	0.223	5-hydroxytryptamine receptor 5A
3	SNCB	9.82E+12	0.077	synuclein beta
4	GRIN1	9.80E+12	0.495	glutamate ionotropic receptor NMDA type subunit 1
5	SNAP25	9.67E+12	0.104	synaptosome associated protein 25
6	GABRG2	9.58E+12	0.692	gamma-aminobutyric acid type A receptor subunit gamma2
7	HPCA	9.57E+12	**0.036**	hippocalcin
8	CPNE6	9.49E+12	0.200	copine 6
9	GABRD	9.39E+12	**0.013**	gamma-aminobutyric acid type A receptor subunit delta
10	ATP2B3	9.23E+12	0.874	ATPase plasma membrane Ca2+ transporting 3

Supplementary Table 3. Baseline characteristics of the selected GBM patients

Sex	Age (y)	OS (days)	KPS
Male	60	80	80
Male	69	106	90
Female	67	135	90
Male	70	157	80
Male	63	175	80
Male	27	223	80
Male	47	226	90
Male	45	243	90
Female	71	257	80
Female	68	348	90
Female	67	378	90
Male	54	405	90
Female	63	440	80
Female	53	447	90
Female	48	462	90
Male	33	483	90
Male	68	503	90
Female	53	562	90
Male	42	625	90
Male	52	704	90
